# MicroRNA-29b Suppresses Inflammation and Protects Blood-Brain Barrier Integrity in Ischemic Stroke

**DOI:** 10.1155/2022/1755416

**Published:** 2022-08-23

**Authors:** Xiaoqing Ma, Ho Jun Yun, Kenneth Elkin, Yunliang Guo, Yuchuan Ding, Guangwen Li

**Affiliations:** ^1^Department of Neurology, The Affiliated Hospital of Qingdao University, No. 16 Jiangsu Road, Qingdao, China; ^2^Institute of Integrative Medicine, Qingdao University, No. 308 Ningxia Road, Qingdao 266071, China; ^3^Wayne State University School of Medicine, Detroit, MI, USA; ^4^Department of Neurology, Institute of Cerebrovascular Diseases, The Affiliated Hospital of Qingdao University, Qingdao 266000, China; ^5^Department of Neurosurgery, Wayne State University School of Medicine, Detroit, MI, USA

## Abstract

**Objectives:**

Following cerebral ischemia, microRNA- (miR-) 29b in circulating blood is downregulated. This study investigates the underlying mechanism and implications of miR-29b in leukocyte induction.

**Methods:**

miR-29b from stroke patients and rats with middle cerebral artery occlusion (MCAO) were assessed using real-time polymerase chain reaction (PCR). miR-29b agomir was used to increase miR-29b expression in leukocytes via intravenous injection. C1q and tumor necrosis factor (C1QTNF) 6, interleukin- (IL-) 1*β*, zonula occludens- (ZO-) 1, occludin, and ischemic outcomes were assessed in MCAO rats. Additionally, hCMEC/D3 cells were subjected to oxygen–glucose deprivation (OGD) and cocultured with HL-60 cells.

**Results:**

miR-29b levels in neutrophils were found to be significantly lower in stroke patients compared with healthy controls, which may indicate its high diagnostic sensitivity and specificity for stroke. Moreover, miR-29b levels in leukocytes showed a negative correlation with National Institute of Health Stroke Scale (NIHSS) scores and C1QTNF6 levels. In MCAO rats, miR-29b overexpression reduced brain infarct volume and brain edema, decreasing IL-1*β* levels in leukocytes and in the brain 24 hours poststroke. miR-29b attenuated IL-1*β* expression via C1QTNF6 inhibition, leading to decreased blood-brain barrier (BBB) disruption and leukocyte infiltration. Moreover, miR-29b overexpression in HL-60 cells downregulated OGD-induced hCMEC/D3 cell apoptosis and increased ZO-1 and occludin levels in vitro.

**Conclusion:**

Leukocytic miR-29b attenuates inflammatory response by augmenting BBB integrity through C1QTNF6, suggesting a novel miR-29b-based therapeutic therapy for ischemic stroke.

## 1. Introduction

Ischemic stroke is one of the most common causes of disability and death in adults worldwide [1, 2]. The pathophysiology of ischemic stroke is characterized by decreased blood flow that reduces delivery of oxygen and other essential nutrients to the brain [3]. Intensive preclinical research has demonstrated various mechanisms which underlie ischemic brain injury, two of which are the inflammatory response and BBB disruption [4]. BBB disruption facilitates secondary brain injury and increases the risk of hemorrhage transformation, heavily influencing the prognosis of ischemic stroke [5].

There is now strong evidence that BBB disruption further contributes to infiltration of various peripheral immune cells [6]. Leukocytes are the most common immune cell infiltrating to the brain after ischemia, which then release inflammatory factors, such as IL-1*β*, to promote their own migration into the central nervous system (CNS) [6]. The infiltration of leukocytes is, thus, a vicious positive feedback loop which further disrupts the BBB after ischemic injury [6].

Experimental research suggests that microRNAs (miRNAs, miRs) in peripheral leukocytes exhibit altered expression after cerebral ischemic injury [7, 8]. miRNAs are short noncoding RNAs that regulate various biological and pathological processes, including modulating multiple messenger RNAs (mRNAs) [8]. Among these miRNAs, miR-29b is most significantly downregulated at an early stage after brain ischemia [9]. Downregulation of miR-29b has previously been reported with an unfavorable prognosis, underscoring the need to investigate miR-29's functional relevance [9].

Recent studies have shown important roles of miR-29b in inflammatory process. One study explains that C1QTNF6 mRNA could be modulated by miR-29b, which was originally identified in the brain and peripheral inflammatory cells [10]. Emerging evidence demonstrates that C1QTNF6 is involved in a variety of physiological processes, namely, the inflammatory reaction [11]. miR-29b is found to act as a novel modulator of inflammatory responses by targeting the C1QTNF6 signaling pathway in human bronchial epithelial cells [11]. Aberrant circulating expression of C1QTNF6 has been found in ischemic patients [12]. In polycystic ovary syndrome, C1QTNF6 upregulates serum levels of proinflammatory factors, such as C-reactive protein (CRP), interleukin 6 (IL-6), and tumor necrosis factor-*α* (TNF-*α*) [13]. Moreover, miR-29b has been found to modulate inflammatory responses by targeting the C1QTNF6 in the lung of mice exposed to ambient particulates, promoting the expression of IL-1*β*, IL-6, and IL-8 [11].

Considering the pathological role of inflammation in ischemic stroke and the abnormal expression of miR-29b and C1QTNF6 in leukocytes of ischemic stroke patients, this study hypothesizes that miR-29b in peripheral leukocytes participates in the inflammatory response by targeting C1QTNF6 and degrading the integrity of the BBB after ischemic stroke.

## 2. Materials and Methods

### 2.1. Study Subjects

This study was registered in ClinicalTrials.gov (NCT03577093). The methods conducted by Li et al. were followed with 60 consecutive acute ischemic stroke patients and 40 healthy controls from Xuanwu Hospital [12]. There was no statistically significant differences in age, gender, and risk factors (hypertension, diabetes, or hypercholesterolemia) between two groups. Neutrophils from the ischemic stroke patients were collected within 6 hours after stroke onset. Ischemic stroke was diagnosed by two neurologists based on clinical history, laboratory findings, neurological deficits on physical exam, and diffusion-weighted magnetic resonance imaging (MRI) results. Real-time polymerase chain reaction (RT-PCR) was performed to measure miR-29b and C1QTNF6 levels in the neutrophils.

### 2.2. Middle Cerebral Artery Occlusion and Groups

Male Sprague-Dawley rats were purchased from Vital River Laboratory Animal Technology Co. (Beijing, China) and reared in the SPF animal husbandry center of Qingdao University. Adult rats weighing 250-280 g were divided into three groups (*n* = 12): sham, MCAO+control, and MCAO+miR-29b agomir. After inducing anesthesia with 1.5% isoflurane, middle cerebral artery occlusion (MCAO) surgery was performed 3 days after intravenous injection of either 100 *μ*L miR-29b agomir or NS control (NC) with 32 *μ*L transfection reagent. MCAO was performed following the methods of Li et al. [12]. The sequence of miR-29b was 5′-UAGCACCAUUUGAAAUCAGUGUU-3′. The rats in the sham group underwent the same anesthesia and surgical procedures without MCAO.

### 2.3. Blood Collection and Separation of Leukocytes

Venous blood was sampled from healthy volunteers, ischemic stroke patients within 6 hours of stroke onset, and rats in 24 hours after MCAO. The blood samples were collected into vacuum tubes with EDTA anticoagulant. Plasma was separated by centrifugation, and the neutrophils and leukocytes were separated as performed in the past study by the authors of this study [7].

### 2.4. Assessment of Neurological Deficits

At 24-hour postreperfusion, neurological deficits were assessed using the Zea Longa 5-point scoring method by a blinded investigator. This was performed as described in the past study conducted by the authors of this study [14].

### 2.5. 2,3,5-Triphenyl-2H-tetrazolium Chloride Staining

After 24 hours of reperfusion, the rats were sacrificed. The brains were quickly removed, cut into 2.0 mm coronal slices, and incubated in a 2% 2,3,5-triphenyl-2H-tetrazolium chloride (TTC) solution for 15 min at 37°C in a dark room [12]. Normal brain tissues were stained red, whereas infarcted tissues were pale gray. The infarction and edema volume were calculated and statistically analyzed by ImageJ and SPSS software. The infarct volume and edema were calculated using the following formulas: Brain infarct volume (%) = ((contralateral hemisphere area − noninfarcted region in the ipsilateral hemisphere)/contralateral hemisphere area) × 100% and Brain edema (%) = ((ipsilateral hemisphere volume − contralateral hemisphere volume)/contralateral hemisphere volume) × 100%.

### 2.6. Western Blot Assay

The ischemic brain tissues, leukocytes of the rats, and cultured cells were assessed by western blotting. Total proteins were extracted using Total Protein Extraction Kit, and protein concentrations were measured with BCA protein assay kit. The extracted proteins were transferred to polyvinylidene fluoride membranes and blocked in 5% fat-free milk powder for 1 hour at room temperature. Primary antibodies were used for occludin, C1QTNF6, *β*-actin (Abcam), IL-1*β* (Catalog), ZO-1 (arigo), and caspase-3 (Abcam and Affinity Biosciences). The membranes were washed with PBST, followed by incubation with horseradish peroxide-conjugated IgG secondary antibody at room temperature. Immunoreactive bands were acquired using a chemiluminescence kit in a dark room. The relative quantity of each band was calculated using AlphaEaseFC v4.0 software and normalized to that of loading controls. All experiments were repeated three times or more.

### 2.7. Real-Time Polymerase Chain Reaction

Neutrophils of stroke patients and healthy controls were collected from venous blood samples. The ipsilateral brain tissues and leukocytes of MCAO rats were isolated and collected 24 hours after reperfusion. RT-PCR was performed to assess miR-29b expression in neutrophils, leukocytes, and cerebral tissues. Total RNA was extracted and reverse-synthesized into cDNA using oligo-d(T) primers and SuperScript III/RNaseOUT Enzyme Mix (Invitrogen, Carlsbad, USA). For miR quantification, total RNA was purified using the RNeasy Mini Kit (Qiagen, Gaithersburg, USA). miRNA abundance was assessed by RT-PCR using All-in-One miRNA RT-PCR Reagent Kits. miR-29b primers for human neutrophils were 5′GGGTAGCACCATTTGAAATCA3′ and 5′GTGCGTGTCGTGGAGTCG3′. miR-29b primers for rat leukocyte were 5′CTCAACTGGTGTCGTGGAGTCGGCAATTCAGTTGAG3′ and 5′ACACTCCAGCTGGGTAGCACCATTTGAAATC3′. C1QTNF6 primers for human neutrophils were 5′TCAGTCCCTTCCACCAAA3′ and 5′ACCTTGATAAAGCCTGGAGA3′. C1QTNF6 primers for rat were 5′GTTCGGGGTCTGTGAGTTGAG3′ and 5′CTTTCAGGATGGTGATGTTGATGTA3′. Relative gene expression was calculated via the 2^-△△CT^ method, normalized, and expressed as fold change relative to U6.

### 2.8. NeuN/TUNEL Staining

The brains were removed and fixed in 4% paraformaldehyde solution for 48 hours, followed by dehydration in 30% sucrose solution at 4°C for 72 hours. The brain tissue was then cut into 20 *μ*m coronal slices on a cryostat vibratome and stored at -20°C. Before staining, the frozen slices were incubated with PBS containing 0.3% Triton X-100 and 10% goat serum for 2 hours. The slices were incubated with primary antibodies (NeuN, Millipore) in a humidified container for 12 hours at 4°C and then with fluorescent-conjugated secondary IgG antibodies for 1 hour at room temperature. 4′,6-Diamidino-2-phenylindole (DAPI) was then used to label cell nuclei. The fluorescence signals were obtained using an Olympus Fluoview FV1000 microscope (Olympus, Japan). The mean number of cells positive for NeuN/TUNEL staining was calculated in the region of the ischemic cortex.

### 2.9. ELISA

Plasma from MCAO rats was collected 24 hours following reperfusion. Approximately 200 *μ*L of plasma was prepared with ice-cold phosphate-buffered solution. The concentrations of IL-1*β* were determined using a commercial ELISA kit (Jiangsu Jingmei Biological Technology) according to the manufacturer's instructions.

### 2.10. Cell Culture, Transfection, and OGD Treatment

HL-60 cells were cultured in 1640 RPMI medium containing 10% heat-inactivated FBS, 100 U/mL penicillin, and 100 mg/mL streptomycin and incubated in a humidified incubator at 37°C with 5% CO_2_ at 37°C. HL-60 cells were transfected with a mixture of miR-29b agomir/control and Lipofectamine RNAiMAX (GenePharma) before a further 24-hour incubation in a humidified incubator. Human brain capillary endothelial cells (hCMEC/D3) were cultured in EBM-2 medium supplemented with vascular endothelial growth factor (VEGF), insulin-like growth factor-1 (IGF-1), epidermal growth factor (EGF), basic FGF, hydrocortisone, ascorbate, penicillin-streptomycin, and 2.5% FCS. hCMEC/D3 in the OGD group was kept in an ischemia-mimetic solution and kept for 2.5 hours at 37°C in a hypoxic incubator chamber filled with 94.5% N_2_, 0.5% O_2_, and 5% CO_2_. hCMEC/D3 was then transferred to normal culture medium for 24 hours and kept at 37°C in an incubator with 5% CO_2_ for reoxygenation. The hCMEC/D3 cells were divided into three groups: sham+control-HL-60 group, OGD+control-HL-60 group, and OGD+miR-29b agomir-HL-60 group (20 nM).

### 2.11. Coculture

HL-60 cells and hCMEC/D3 were cocultivated with a transwell coculture system. HL-60 cells at a density of 10 × 10^5^/well were seeded on the apical side of transwell membranes (0.4 *μ*m pore size, Corning, NY, USA) and cultivated with supplemented medium. The hCMEC/D3 were seeded to the basal side and were further cultivated in the static monoculture model. The HL-60 cells and hCMEC/D3 cells were harvested at 24 hours after coculturing.

### 2.12. Flow Cytometry

Apoptosis of hCMEC/D3 was detected by flow cytometry analysis using Dead Cell Apoptosis Kit with Annexin V Alexa Fluor 488. hCMEC/D3 were harvested and washed with cold PBS twice and then incubated with Annexin V-FITC mixed with propidium iodide for 10 minutes in a dark room. Cellular fluorescence was assessed by flow cytometry analysis (CytoFLEX S, Beckman, China).

### 2.13. Statistical Analysis

Data were analyzed using SPSS version 17.0 (SPSS, Chicago, IL) and are expressed as mean ± standard deviation (SD). *t* test was performed for two-group comparisons. One-way analysis of variance (ANOVA) with Tukey's comparison analysis was performed to compare several quantitative variables. Pearson's correlation test was used to assess the correlation between two variables. Statistical significance was noted if *P* < 0.05.

## 3. Results

### 3.1. miR-29b Expression in Neutrophils Is Downregulated in Ischemic Stroke

**Figure 1:**
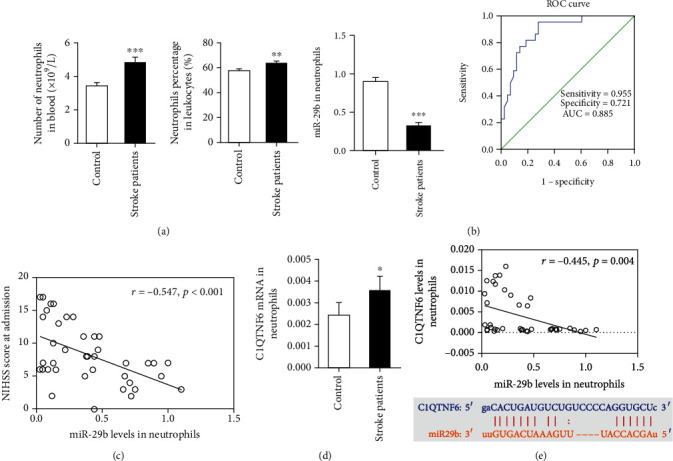
Neutrophilic miR-29b levels are significantly lower in stroke patients and show strong diagnostic value. (a) The number and percentage of neutrophils in stroke patients. (b) miR-29b levels in neutrophils of stroke patients. Receiver operating characteristic (ROC) curve of miR-29b in diagnosing ischemic stroke. (c) Correlation between miR-29b expression and National Institute of Health Stroke Scale (NIHSS) score at admission. (d) C1QTNF6 mRNA levels in neutrophils of ischemic stroke patients. (e) Correlation between miR-29b and C1QTNF6 expression at admission. The predicted binding sites between miR-29b and C1QTNF6. *N* = 60 in stroke group and *N* = 40 in control group. ^∗^*P* < 0.05, ^∗∗^*P* < 0.01, and ^∗∗∗^*P* < 0.001 vs. control group. AUC indicates area under the ROC curve.

The number of neutrophils in circulating blood of acute ischemic stroke patients significantly increased compared to that of health controls (Figure 1(a); *P* < 0.05). Additionally, miR-29b expression shown by RT-PCR demonstrated that miR-29b levels in neutrophils significantly decreased after ischemic stroke (Figure 1(b); *P* < 0.05).

A cut-off point of 0.575 was used to differentiate stroke patients from healthy controls with a sensitivity of 0.955 and a specificity of 0.721 (Figure 1(b)). The area under the ROC curve (AUC) of miR-29b in neutrophils was 0.885, indicating that it had a high diagnostic value for ischemic stroke. A negative linear correlation was observed between miR-29b levels in neutrophils and NIHSS score at admission (Figure 1(c); *R* = −0.547, *P* < 0.001).

### 3.2. C1QTNF6 Is Upregulated and Negatively Associated with miR-29b Levels

C1QTNF6 has been found to be the molecular target of miR-29b [10]. In this study, mRNA expression of C1QTNF6 in neutrophils following ischemic stroke significantly increased compared to that of healthy controls (Figure 1(d); *P* < 0.05). miR-29b levels were found to be negatively correlated with C1QTNF6 mRNA in neutrophils as demonstrated by the bioinformatics software TargetScan and miRmap predicting the putative miR-29b binding sites of C1QTNF6 mRNA (Figure 1(e); *R* = −0.445, *P* = 0.004).

### 3.3. miR-29b Is Downregulated in Leukocytes and the Brain of MCAO Rats

**Figure 2:**
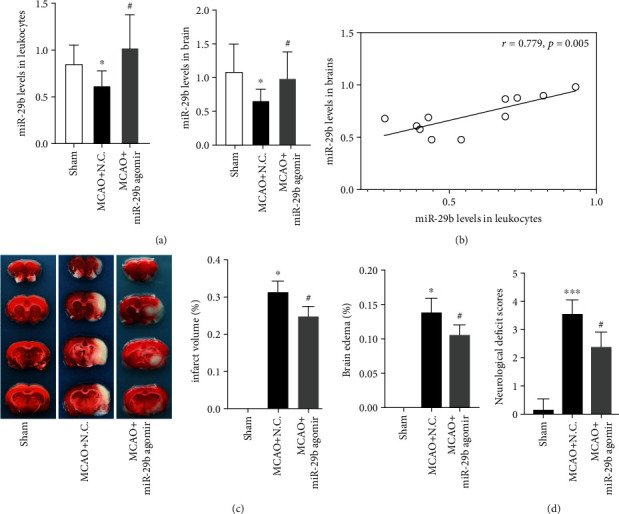
Changes of miR-29b expression and subsequent effects on cerebral injury and neurological deficits in middle cerebral artery occlusion (MCAO) rats. (a) The expression of miR-29b in leukocytes and the ischemic brain. *N* = 9. (b) The correlation between leukocyte miR-29b and brain miR-29b levels. *N* = 9. (c) Cerebral infarct volume and edema evaluated by TTC staining of coronal brain sections. *N* = 12. (d) Effects of miR-29b on neurological function deficits at 24-hour poststroke. ^∗^*P* < 0.05 and ^∗∗∗^*P* < 0.001 vs. sham group. ^#^*P* < 0.05 vs. MCAO+NC group.

The expression of miR-29b in leukocytes and the brain tissues were measured by RT-PCR. miR-29b was remarkably downregulated in both (Figure 2(a); *P* < 0.05). To study the relationship between leukocytic and cerebral miR-29b levels, miR-29b expression of leukocytes and brain tissues from each rat was assessed. Correlation analysis showed that leukocytic miR-29b expression was positively correlated with cerebral miR-29b level (Figure 2(b); *R* = 0.779, *P* = 0.005).

### 3.4. MicroRNA-29b Overexpression Mitigates Cerebral Injury in Middle Cerebral Artery Occlusion Rats

To evaluate the role of miR-29b, rats were injected intravenously with miR-29b agomir three days before MCAO. miR-29b expression in leukocytes and the brain tissues successfully increased after miR-29b agomir injection, suggesting proper transduction (Figure 2(a); *P* < 0.05). Brain infarct volume and edema were measured to assess the effect of miR-29b overexpression. TTC staining showed that the infarct volume was significantly decreased in the miR-29b agomir group compared to the control group (Figure 2(c); *P* < 0.05). Similarly, edema formation of ipsilateral hemisphere in the miR-29b agomir group was significantly lower than the control group (Figure 2(c); *P* < 0.05). In addition, neurological deficits in the miR-29b agomir group significantly improved when compared to the control group (Figure 2(d); *P* < 0.05).

### 3.5. miR-29b Decreases Neuronal Apoptosis in MCAO Rats

**Figure 3:**
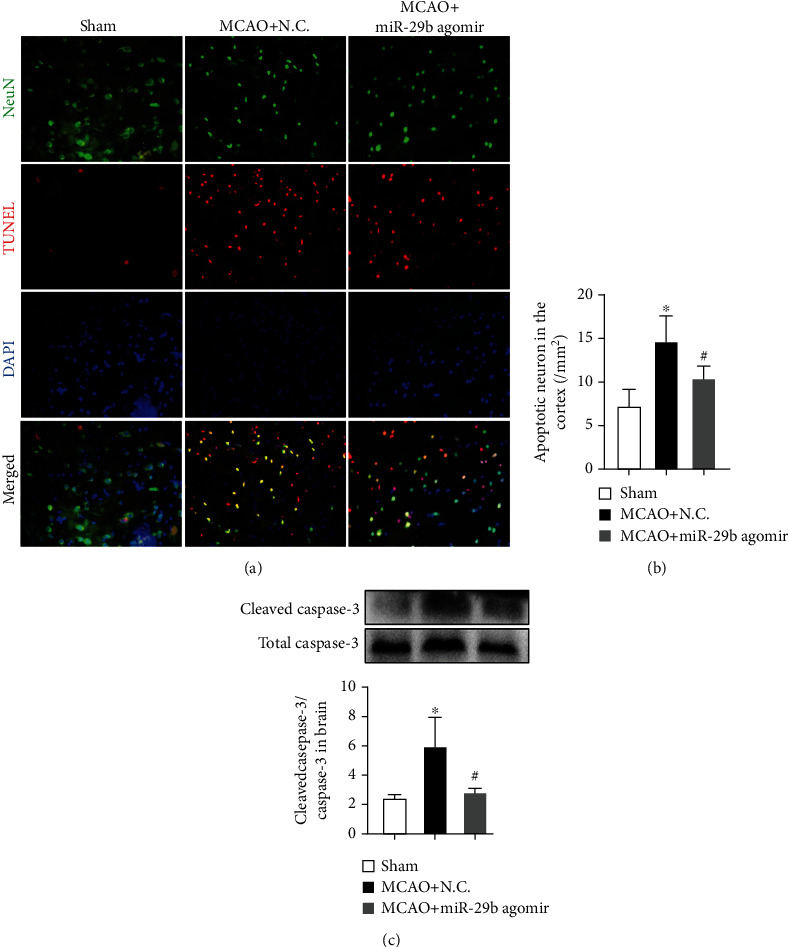
miR-29b inhibited the neuronal apoptosis after MCAO in rats. (a and b) Neuronal apoptosis in the ipsilateral cortex detected by NeuN/TUNEL immunofluorescence double staining. *N* = 6. (c) Immunoblot of caspase-3 in the ipsilateral brain of MCAO rats. *N* = 6. ^∗^*P* < 0.05 vs. sham group. ^#^*P* < 0.05 vs. MCAO+NC group.

Immunofluorescence staining was used to analyze the degree of apoptotic neurons in the cortex. MCAO increased the number of TUNEL/NeuN-positive cells in the cortex compared to sham rats, which was mitigated by miR-29b agomir treatment (Figures 3(a) and 3(b); *F* = 30.37, *P* < 0.05). Additionally, cleavage of caspase 3, an indicator of apoptosis, was remarkably decreased in the miR-29b agomir group compared to the MCAO group (Figure 3(c); *F* = 10.42, *P* < 0.05).

### 3.6. miR-29b Attenuates IL-1*β* Expression by Inhibiting C1QTNF6 in MCAO Rats

**Figure 4:**
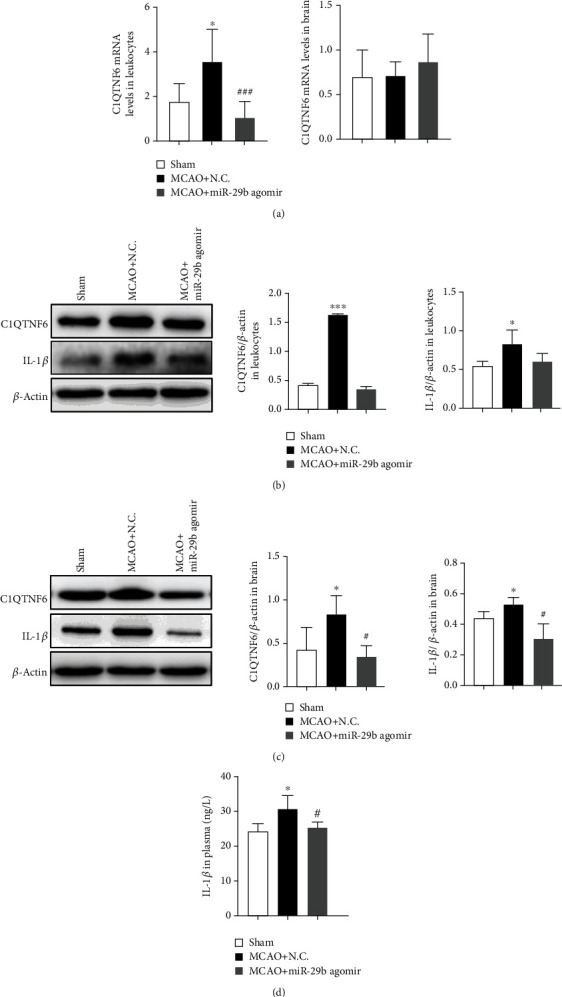
miR-29b downregulated C1QTNF6 expression in leukocytes of MCAO rats. (a) C1QTNF6 mRNA levels in leukocytes and the brain. *N* = 6. (b) Protein expression of C1QTNF6 and interleukin- (IL-) 1*β* in leukocytes assessed by Western blot. *N* = 6. (c) Protein levels of C1QTNF6 and IL-1*β* in the brain measured by Western blot. *N* = 6. (d) IL-1*β* expression in plasma measured at 24 h after MCAO injury. *N* = 12. ^∗^*P* < 0.05 and ^∗∗∗^*P* < 0.001 vs. sham group. ^#^*P* < 0.05 and ^###^*P* < 0.001 vs. MCAO+NC group.

To assess the effect of miR-29b on C1QTNF6 levels in MCAO rats, C1QTNF6 mRNA and protein in MCAO rats were measured. C1QTNF6 mRNA levels in leukocytes were significantly higher in MCAO rats than the sham control, and this increase was mitigated by miR-29b agomir (Figure 4(a); *P* < 0.05). C1QTNF6 protein expression in leukocytes and in the brain was significantly increased compared to the sham control, and this change was, again, attenuated by miR-29b agomir (Figures 4(b) and 4(c); *P* < 0.05). However, C1QTNF6 mRNA expression in the brain was not changed significantly by MCAO or miR-29b agomir treatment (Figure 4(a); *P* > 0.05). IL-1*β* expression in leukocytes, the brain, and plasma was significantly increased after MCAO injury and reduced by miR-29b agomir (Figures 4(b)–4(d); *P* < 0.05).

### 3.7. miR-29b Attenuates Blood-Brain Barrier Disruption in Ischemic Rats

**Figure 5:**
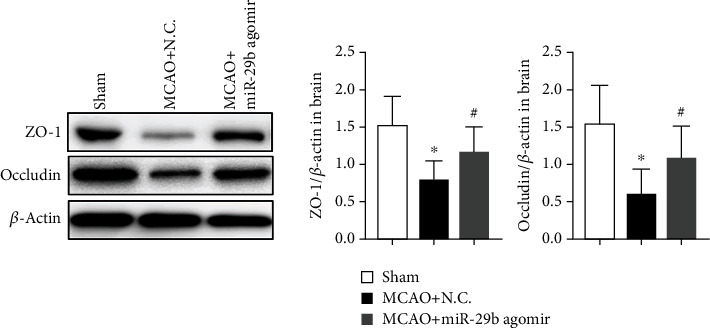
Protein expression of ZO-1 and occludin in the brain measured by Western blot. *N* = 6. ^∗^*P* < 0.05 vs. sham group; ^#^P < 0.05 vs. MCAO+NC group.

As leukocyte migration is known to damage BBB integrity after ischemic stroke [15], expression of ZO-1 and occludin in the cerebrum was measured by Western blot. The expression of ZO-1 and occludin in the brain was decreased by MCAO injury compared to the sham control. The decreased expression of ZO-1 and occludin was reversed by miR-29b agomir (Figure 5; *P* < 0.05).

### 3.8. miR-29b in HL-60 Cells Downregulates hCMEC/D3 Apoptosis and Upregulates ZO-1 and Occludin Levels In Vitro

**Figure 6:**
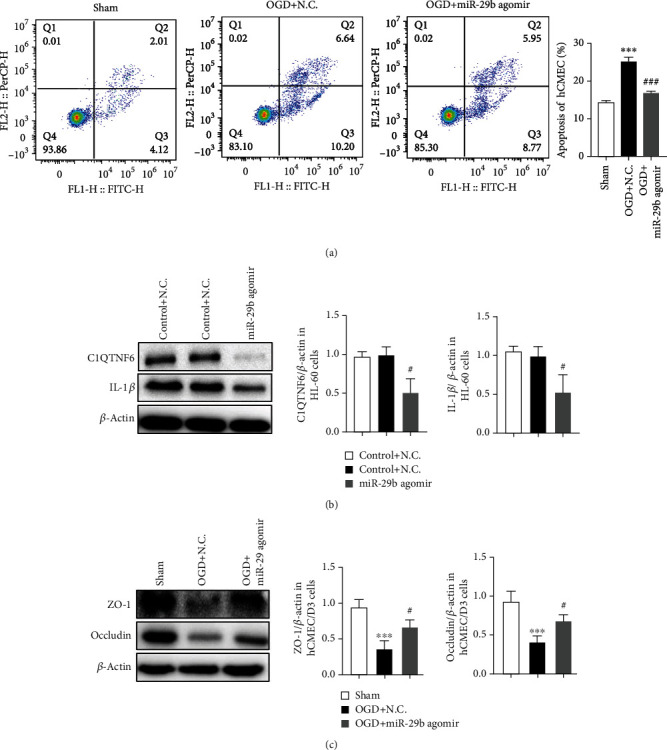
miR-29b in leukocytes regulates zonula occludens- (ZO-) 1 and occludin protein expression in oxygen–glucose deprivation (OGD) hCMEC/D3 cells. (a) Apoptosis rate of hCMEC/D3 cells cocultured with HL-60 cells treated with either miR-29b agomir or control agent. *N* = 6. (b) Protein expression of C1QTNF6 and IL-1*β* in the HL-60 cells assessed by Western blot. *N* = 6. (c) Protein levels of ZO-1 and occludin in hCMEC/D3 cells measured by Western blot. *N* = 6. ^∗∗∗^*P* < 0.001 vs. sham group; ^#^*P* < 0.05 vs. OGD group; ^###^*P* < 0.001 vs. OGD+NC group.

To assess the protective effect of miR-29b in leukocytes on BBB integrity, the OGD-treated-hCMEC/D3 cells were cocultured with HL-60 cells which were treated with either miR-29b agomir or control agent. Flow cytometry assay showed that OGD induced more apoptosis on hCMEC/D3 cells than the sham group. miR-29b agomir also decreased OGD-induced apoptosis of hCMEC/D3 cells (Figure 6(a); *F* = 17.6, *P* < 0.05). The expression of C1QTNF6 and IL-1*β* in HL-60 cells was decreased after miR-29b agomir treatment (Figure 6(b); *P* < 0.05). ZO-1 and occludin expressions in hCMEC/D3 cells were measured after cocultured with HL-60 cells. The expression of ZO-1 and occludin in hCMEC/D3 cells was reduced with OGD (Figure 6(c); *P* < 0.05). The reduced expression of ZO-1 and occludin was reversed when treated with miR-29b agomir (Figure 6(c); *P* < 0.05). This suggests that miR-29b has a protective role for BBB integrity by inhibiting endothelial cells apoptosis and increasing ZO-1 and occludin levels.

## 4. Discussions

Studies have noted that miRNA expression in peripheral leukocytes after ischemic stroke is altered, and some miRNAs may be utilized as ischemic stroke biomarkers [8, 16]. This is the first report to investigate the clinical value of miR-29b in peripheral neutrophils from patients with acute ischemic stroke. There are two important implications from the results of this research. First, a novel circulating biomarker, such as miR-29b in leukocytes, could help diagnose and predict the severity of stroke. Secondly, miR-29b plays imperative roles as a downregulatory of C1QTNF6 in leukocytes, leading to reduction in ischemia-induced inflammatory response, BBB disruption, and ultimately infarct volume.

Currently, there are no serum biomarkers with clinical utility to diagnose ischemic stroke. Studies have shown that miR-29b levels are decreased in stroke patients and negatively associated with NIHSS scores and higher infarct volume [17]. In this study, miR-29b expression in leukocytes showed a diagnostic value for acute ischemic stroke. miR-29b levels in neutrophils were remarkably decreased in the early stage of stroke. Additionally, low miR-29b levels in neutrophils were correlated with neurological deficits at admission.

Neutrophilic miR-29b may play a crucial pathologic role in ischemic stroke. This study demonstrated that miR-29b downregulation occurs simultaneously in leukocytes and the brain of MCAO rats [17]. A positive correlation was observed between leukocyte miR-29b levels and ischemic brain tissue, suggesting that leukocytic miR-29b may represent the expression of miR-29b in the brain, hence the severity of cerebral injury.

miR-29b has been found to promote IL-1*β* expression by inhibiting C1QTNF6 levels in human bronchial epithelial cells [11]. This study showed abnormally high expression of C1QTNF6 in leukocytes of patients with ischemic stroke and in MCAO rats. This finding implies that C1QTNF6 can be regulated directly by miR-29b. Li et al. found that C1QTNF6 rescued OGD-induced PC12 cells and reduced the secretion of inflammatory reactants (i.e., TNF-*α* and IL-1*β*) [18]. CIQTNF6 also increased IL-10 expression in the macrophage and modulates the inflammatory response [19]. Although inconsistent with previous studies, this study observed that C1QTNF6, as the target gene of miR-29b, increased the expression of IL-1*β* in leukocytes of MCAO rats and HL-60 cells. This discrepancy may have resulted from differences in model systems, timing of sample analysis, and the cells or tissues selected for analysis.

Infiltrating leukocytes worsen ischemic injury, whereas inhibition of leukocytic infiltration ultimately reduces ischemic volume [20]. C1QTNF6 overexpression was shown to promote MCAO-induced IL-1*β* expression of leukocytes, which attracts leukocytes to ischemic brain [21, 22]. The infiltrating neutrophils release cytokines and chemokines worsening the brain injury [23, 24]. Therefore, regulating neutrophil infiltration may be an important factor in modulating secondary damage from cerebral ischemia.

This study showed that MCAO injury increases the infiltration of peripheral leukocytes, while miR-29b treatment decreases the infiltration. miR-29b decreased C1QTNF6 expression and IL-1*β* levels in leukocytes, which could inhibit neutrophil migration into the brain parenchyma. Huang et al. have suggested that the infiltration of peripheral leukocytes and proinflammatory mediators from these leukocytes may disrupt the BBB during and after ischemic stroke [25]. The present study demonstrated that miR-29b overexpression in leukocytes reduces cerebral infarct volume, edema formation, and neuronal death potentially through inhibition of endothelial cell apoptosis and increased tight junction protein formation of microvascular endothelium.

## 5. Conclusions

miR-29b of peripheral leukocytes can be an attractive therapeutic target the treatment of ischemic stroke and a molecular marker to assess the severity of stroke. Endogenous protective mechanism of miR-29b against brain injury is partly due to the BBB protection by decreasing C1QTNF6 and IL-1*β* expression in leukocytes. Nevertheless, ischemic stroke involves complicated pathologic process, and inhibiting the inflammatory response and protecting BBB integrity by miR-29b overexpression may not be sufficient for complete recovery. Further studies are warranted to carefully examine miR-29b in ischemic stroke.

## Data Availability

The datasets used and analyzed during the current study are available from the corresponding author on reasonable request.
